# Successful Treatment of Respiratory Failure in a Patient with Prader-Willi Syndrome with Noninvasive Ventilation with AVAPS

**DOI:** 10.1155/2023/9925144

**Published:** 2023-04-18

**Authors:** Nauras Hwig, Montserrat Diaz-Abad, Victor T. Peng, Jennifer Y. So, Anayansi Lasso-Pirot

**Affiliations:** ^1^Sleep Disorders Center, University of Maryland Medical Center, Baltimore, Maryland, USA; ^2^Department of Medicine, Division of Pulmonary and Critical Care Medicine, University of Maryland School of Medicine, Baltimore, Maryland, USA; ^3^Department of Pediatrics, Division of Pediatric Pulmonology, University of Maryland School of Medicine, Baltimore, Maryland, USA

## Abstract

Prader-Willi syndrome (PWS) is the most prevalent syndromic form of obesity, which starts during early childhood in the setting of hyperphagia. Due to the development of obesity, there is a high prevalence of obstructive sleep apnea (OSA) among these patients. This case report presents a patient with PWS with morbid obesity, severe OSA, and obesity hypoventilation syndrome admitted to the hospital for hypoxemic and hypercapnic respiratory failure. Noninvasive ventilation (NIV) with average volume-assured pressure support, a newer NIV modality, was used successfully to treat this patient, achieving major clinical and gas exchange improvement both during the hospitalization and long term after discharge.

## 1. Introduction

Prader-Willi syndrome (PWS) has a prevalence of about 1/15,000 people and is the most common syndromic form of obesity. The syndrome is caused by lack of expression of the long arm on chromosome 15, specifically 15q11.2-q13, with 65–75% of the cases associated with paternal deletion of this chromosomal region [1].

PWS is characterized by a multitude of features that occur from early childhood to adolescence, with one of the primary findings being the development of obesity during early childhood in the setting of hyperphagia. Because of this, there is a high prevalence of obstructive sleep apnea (OSA) among these patients [1, 2]. Adenotonsillectomy is the primary treatment for OSA in pediatric patients; if unsuccessful, continuous positive airway pressure (CPAP) therapy is indicated [3, 4]. If CPAP and noninvasive ventilation (NIV) fail, tracheostomy is a treatment option of last resort. This case report presents a patient with PWS with morbid obesity, severe OSA, and obesity hypoventilation syndrome (OHS) who was admitted for hypoxemic and hypercapnic respiratory failure. NIV with average volume-assured pressure support with auto-titrating end expiratory airway pressure (EPAP), (AVAPS-AE), a newer NIV modality, successfully treated the hypoventilation during the hospitalization and after discharge.

## 2. Case Presentation

A 6-year-old boy with past medical history of PWS, hypotonia, mild developmental delay, and early onset thoracic scoliosis treated with posterior spinal growing rod placement presented with a 2-year history of loud snoring. He underwent in-laboratory polysomnography (PSG), which showed severe OSA, with apnea-hypopnea index (AHI) 133.7 events/hour. The patient was admitted to the Pediatric Intensive Care Unit (PICU) and placed on CPAP 6 cm H_2_O with clinical improvement. He was started on home CPAP and underwent an in-laboratory CPAP titration in which the AHI improved to 1.2 events/hour with nasal CPAP 14 cm H_2_O. After that, the patient underwent surgical intervention with a combined uvulopalatopharyngoplasty and adenotonsillectomy. Postoperative PSG showed improved but persistent severe OSA with residual AHI 37 events/hour. The patient did not attend postsurgery CPAP titration study and was noted to have poor CPAP compliance over the next 2 years, after which he was lost to follow up.

**Figure 1:**
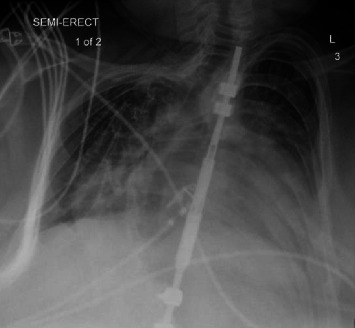
Chest X-ray during Pediatric Intensive Care Unit admission demonstrating enlarged cardiac silhouette, nonspecific bilateral airspace opacities, perihilar interstitial markings, and haziness of the left costophrenic angle. No evidence of major pleural effusion or pneumothorax. Thoracic scoliosis and posterior spinal growing rod are visualized. Suboptimal evaluation related to body habitus.

At age 11, the patient presented to the hospital emergency room with a 2-day history of fatigue, headache, lethargy, sore throat, cough, shortness of breath, abdominal pain, and swelling in the abdomen and lower extremities. His sister had symptoms consistent with an upper respiratory tract infection. On presentation, he had severe hypoxia, SpO_2_ 50% on room air, and was placed on oxygen via non-rebreather mask 15 L/min with improvement in SpO_2_ to 98%. Temperature was 36.8°C, blood pressure 167/95 mm·Hg, heart rate 105 beats/minute, respiratory rate 55 breaths/minute, height 1.42 m, weight 107 kg, and body mass index (BMI) 53.1 kg/m^2^ (>99^th^ percentile). He had no known history of associated cardiac or endocrine disorders and was on no medications at home. On physical exam, the patient was awake, alert, oriented, and in severe respiratory distress. There were nasal flaring, subcoastal retractions, decreased air movement with diffuse coarse rhonchi and crackles on lung auscultation, tachycardia, generalized abdominal tenderness, and 1+ bilateral lower extremity edema. Attempts to transition to oxygen via nasal cannula at 4 L/min were unsuccessful due to oxygen desaturation to 79%. He was switched to high flow nasal cannula (HFNC) at 15 L/min and FiO_2_ 80%. Chest X-ray showed bilateral pulmonary airspace opacities (Figure 1). Admission laboratory results were significant for white blood cell count 20.7 L/mcL, NT-proBNP 1466 pg/mL, troponin 0.08 ng/mL, and venous blood gas (VBG) with pH 7.34, PCO_2_ 73 mm·Hg, and PO_2_ 40 mm·Hg. A complete respiratory viral panel and SARS-CoV-2 testing with polymerase chain reaction were negative. The patient was admitted to the PICU with acute on chronic hypoxemic and hypercapnic respiratory failure in the setting of volume overload, congestive heart failure, and CPAP noncompliance.

The patient continued oxygen via HFNC and was treated with high dose intravenous diuretics (furosemide) for volume overload and replacement therapy with potassium chloride. He also received deep venous thrombosis prophylaxis. Serial troponins peaked to 0.09 ng/mL before decreasing to <0.02 ng/mL. An echocardiogram showed normal biventricular function, with right ventricular pressures estimated to be half of systemic arterial pressures, suggestive of right heart strain and pulmonary hypertension. The patient was treated with daytime HFNC oxygen and nocturnal NIV with bilevel PAP with a Respironics V60 ventilator (Philips Respironics, Murrysville, Pennsylvania, USA), with settings of inspiratory positive airway pressure (IPAP) 12 cm H_2_O and EPAP 6 cm H_2_O, with FiO_2_ 50%. On day 2 of admission the VBG improved, with pH 7.39 and PCO_2_ 63 mm·Hg and the volume status also improved with diuresis. The VBG remained stable on day 3 with pH 7.37 and pCO_2_ 67 mm·Hg, and the patient was transitioned to CPAP 10 cm H_2_O with FiO_2_ 40%. He did not tolerate CPAP that night and later bilevel PAP 16/6 cm H_2_O due to discomfort, increased work of breathing and continued hypercapnia with both, with worsening VBG to pH 7.33 and pCO_2_ 86 mm·Hg on day 4. To avoid intubation and invasive mechanical ventilation, the patient was placed on continuous NIV with AVAPS, a mode of noninvasive ventilation previously used successfully in a pediatric patient at our institution. Initial settings were target tidal volume, (VT) 350 mL (8 mL/kg), EPAP 8 cm H_2_O, IPAP 15–25 cm H_2_O, FiO_2_ 30%, respiratory rate 15 breaths/minute, inspiratory time 1 second, rise time 1. AVAPS was used and well tolerated by the patient and the next day, there was significant improvement in gas exchange, with weaning of FiO_2_ to 21% on the NIV, and VBG showing pH 7.36 and pCO_2_ 41 mm·Hg. Over the course of the next 5 days, the patient's volume and respiratory status continued to improve. He was switched from continuous to nocturnal NIV and daytime oxygen was discontinued. On day 10 of admission, the VBG was pH 7.35 and pCO_2_ 56 mm Hg and weight was 101.7 kg (net loss 5.3 kg). The patient was discharged home that day on nocturnal NIV with a Trilogy ventilator (Philips Respironics) on the following settings: AVAPS-AE, target VT 350 ml, EPAP 6–10 cm H_2_O, pressure support 5–15 cm H_2_O, respiratory rate 15 breaths/minute, inspiratory time 0.8 seconds, rise time 3, and FiO_2_ 21%.

The patient has had a total of four outpatient follow-up visits over 8 months since his discharge and is doing clinically well without hospital readmissions. He has tolerated nocturnal NIV and uses it regularly. His ventilator download data over the most recent 30 days showed excellent compliance, with 93% of days used, with average daily use 6.9 hours, average VT 400 ml, average respiratory rate 21 breaths/minute, and average minute ventilation 8.4 L/min. The patient also lost 4.5 kg during that period. Overall treatment course is focused on maintaining compliance to NIV therapy, as well as weight loss goals, managing symptoms of hyperphagia, and overall treatment of PWS.

## 3. Discussion

This case highlights the potential therapeutic value of NIV with auto-titrating IPAP and EPAP to treat severe OSA with OHS and hypercapnic respiratory failure in patients with PWS. In patients with OSA, surgical intervention is typically the first choice for therapy, and CPAP is recommended for those that fail surgery. In patients with PWS, however, adenotonsillectomy may cause velopharyngeal insufficiency [5], and therefore, PAP therapy may be superior.

Obesity affects 14 million children in the United States [6], and OSA has an estimated prevalence of 1.2% to 5.7% of children [3]. The current mainstay treatment of OSA is adenotonsillectomy and if needed, CPAP therapy [3]. The combination of severe OSA with morbid obesity reduces the benefit of upper airway therapy, and the frequency of perioperative airway complications is also 13% higher (15% vs 2.0%) in severely obese children who undergo tonsillectomy [4]. With the obesity epidemic, rates of treatment failures with surgical intervention and traditional PAP therapy may increase and consideration of alternatives is important. NIV therapy may be a treatment option in severe cases of OSA when other therapies fail to avoid surgical interventions of last resort such as tracheostomy [7].

Patients with PWS are at higher risk for sleep disordered breathing due to various reasons including obesity, craniofacial features, and hypotonia. In fact, the prevalence of OSA in PWS is 80%, which is much greater than in the general pediatric population [8]. Adenotonsillar hypertrophy is a primary contributor to pediatric OSA, but it may also be present as a side effect of growth hormone treatment in PWS. Though the obstructive aspects of sleep apnea may be explained, the etiology of higher prevalence of central sleep apnea in this population is unknown at this time. Patients with PWS have abnormal ventilator control during sleep, higher arousal threshold for hypercapnia, reduced ventilatory response, and limited arousal response to hypoxia. A combination of these factors would indicate a significant risk for sleep-related hypoxia and hypoventilation [5, 8]. In fact, it is common to the PWS phenotype to present with nocturnal hypoventilation, which is not typical for most other patients with OSA, and this may occur without central or obstructive sleep apnea [5]. On NIV with AVAPS-AE, respiratory failure with hypoxemia and hypercapnia can resolve, as in the case of our patient. In addition, this case showcases the potential of maintaining long-term stability as an outpatient with this treatment modality with appropriate compliance, thus avoiding further hospital admissions and other surgical interventions like a tracheostomy.

CPAP therapy is a type of conventional OSA treatment that provides a constant flow of gas at a predetermined pressure [9]. AVAPS is a mode of NIV with IPAP that targets a goal VT based on ideal body weight to treat hypoventilation. The spontaneous respiratory flow is monitored, and the ventilator adjusts the IPAP within a set range to maintain the target VT. AVAPS-AE adds auto-titrating EPAP to treat comorbid OSA [9]. Due to the novelty of the mode and its recent introduction to the pediatric population, there are limited data on AVAPS therapy as a treatment modality for hypoventilation in children, with mostly case reports published despite increasing interest and use over time. We have previously shown AVAPS to be effective in treating very severe OSA and nocturnal hypoventilation in a child both in the sleep laboratory and long term as outpatient avoiding the need for tracheostomy [7]. This modality has also been used successfully in a group of 45 adult patients with OSA who failed a prior CPAP titration [10].

The largest case series of AVAPS use in the pediatric population was published in 2021 and included 19 patients with nocturnal hypoventilation due to multiple medical conditions who had failed a bilevel PAP titration in the sleep laboratory due to persistent hypoventilation [11]. AVAPS titration was shown to be more effective than conventional bilevel PAP titration in decreasing nocturnal transcutaneous CO_2_ levels. This case series included a 12-year-old patient with PWS, the only patient with morbid obesity in the group. To our knowledge, this is the only previous report of use of this NIV modality in PWS. In our case, we document the effectiveness of AVAPS therapy during an inpatient admission for cardiorespiratory failure and for long-term use while outpatient.

In conclusion, we report on the successful use of NIV with AVAPS in treating severe OSA, OHS, and cardiorespiratory failure in a pediatric patient with morbid obesity and PWS, both in inpatient and outpatient settings when other NIV modalities failed. A trial of this NIV modality may be considered in pediatrics as a treatment option in complicated or refractory cases of severe OSA and OHS with associated severe gas exchange abnormalities prior to the use of invasive ventilation. More studies need to be done to clarify the therapeutic role of this NIV modality in these patients.

## Data Availability

No data were made available for this study.
